# Options in the management of tuberculous ureteric stricture

**DOI:** 10.4103/0970-1591.42621

**Published:** 2008

**Authors:** Apul Goel, D. Dalela

**Affiliations:** Department of Urology, CSM Medical University, Lucknow, UP, India

**Keywords:** Tuberculosis, ureter, ureteric stricture

## Abstract

Ureteric stricture is a feared manifestation of genitourinary tuberculosis (TB) with the commonest site being the lower ureter. The purpose of this review is to discuss the management options for this condition. Literature search was done using PubMed and all articles on TB and ureteric stricture were reviewed published between 1990 till September 2007. The exact site and length of stricture must be defined with radioimaging (intravenous urography, retrograde, or antegrade pyelography) and renal function be quantified. The treatment of stricture mostly requires some kind of intervention after a brief period of antituberculous medicines with or without steroids. For uncomplicated/simple strictures (short segment, passable, with renal function >25%, good bladder capacity) endourologic option should be used which usually means double-J stenting with or without balloon dilatation. For complicated/complex strictures (long segment, dense fibrosis, with renal function <20%, small bladder capacity) regular surgical options should be considered which usually means ureteroureterostomy or ureteropyelostomy for upper ureteric strictures, intubated ureterostomy, or transureteroureterostomy for midureteric strictures, psoas hitch/Boari flap for lower ureteric strictures or ileal ureter/autotransplantation for whole length/multiple strictures.

## INTRODUCTION

The World Health Organization estimates that one-third of the world's population is infected with *Mycobacterium tuberculosis* and there are 8-10 million new active cases of tuberculosis (TB) each year.[1] Ureteric stricture following TB is not uncommon with one recent article from China reporting tuberculous ureteric stricture as a cause of hydronephrosis in 11 out of 141 middle-aged and elderly patients.[2] Lower ureteric stricture is seen in about 9% of patients with genitourinary tuberculosis (GUTB).[3]

Management of ureteral stricture poses both a diagnostic dilemma as well as taxes the surgical skills of the reconstructive surgeon. If not properly managed the kidney may be lost. Similarly, the clinician should be careful in declaring the prognosis of these cases as the outcome of ureteral involvement is also dependant on the extent of renal involvement. The purpose of this review is to discuss the investigations and management options for tuberculous ureteric stricture. Literature search was done using PubMed and all articles on TB and ureteric stricture were reviewed published between 1990 till September 2007.

## PATHOLOGY

The commonest site of tuberculous stricture formation is close to ureterovesical junction. Stricture formation is also seen at the level of ureteropelvic junction and less commonly in the middle third of the ureter.[3] Although rare, the entire length of ureter may be involved. The length of stricture varies, but lower ureteric strictures are usually <5 cm in length.[3] While the circular fibrosis gives rise to strictures, the longitudinal fibrosis leads to shortening of ureter, pulling up the orifice as a gaping hole -the so-called ‘golf hole’.[4] Ureteric calcification, albeit a theoretical possibility, is rarely seen clinically.

## DIAGNOSTIC WORKUP

Apart from standard urinary, hematologic, immunologic investigations that are required to establish the diagnosis of GUTB, the specific investigations in a case of ureteric stricture are principally aimed at defining:

- the site, extent, number, and caliber of stricture,
- defining the degree and impact of backpressure changes and/or coassociated tuberculous disease on renal function,
- defining the extent of involvement of urinary bladder and contralateral unit, and
- defining extent of tuberculous involvement in the segment of bowel which may be required for ureteric replacement

**Figure 1:**
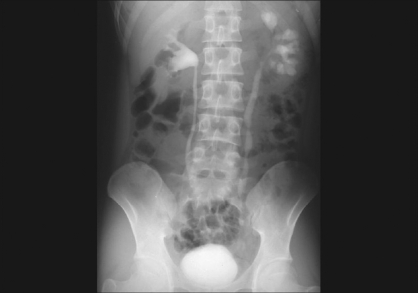
Intravenous urogram showing diseased left kidney and hydroureteronephrosis till the end of mid-ureter

**Figure 2:**
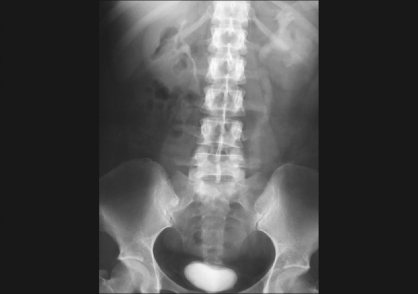
Intravenous urogram showing diseased left kidney with hydroureteronephrosis because of stricture of lower ureter. The bladder is of small capacity

**Figure 3:**
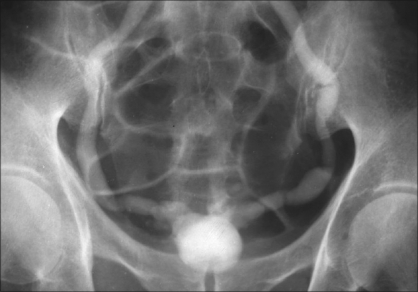
Intravenous urogram showing a ‘thimble’ bladder

The investigative workup must be able to define the morbid anatomy/functional status of entire urinary tract using uroimaging modalities like intravenous urogram [Figures 1 and 2] or retrograde or antegrade ureteropyelogram/magnetic resonance urography in case of poor renal function. Cystography is needed to have an idea about bladder capacity and vesicoureteric reflux [Figure 3]. Computed tomography (CT) urography is now considered the imaging modality of choice for diagnosis and evaluation of GUTB.[3] Three-dimensional image reconstruction is possible and abdominal imaging may be of additional value for defining extent of coassociated retroperitoneal or mesenteric fibrosis and bowel disease. Similarly ultrasonography can be used to monitor the size of kidney during chemotherapy or to monitor the volume of contracted bladder during treatment. The actual quantification of renal functional status of both kidneys by radionuclide scan is of key value in planning definitive treatment and prognosticating the outcome and hence should preferably be done in most cases, especially if the kidney is nonvisualized on intravenous urography (IVU). Another issue that needs to be addressed is to find out as to whether the stricture disease represents old/healed fibrosis or quiescent disease or there is some degree of ongoing active inflammation. One may use nonspecific predictors such as total/differential leukocyte counts, erythrocyte sedimentation rate, antituberculous antibody titers, but more valuable are demonstration of microscopic hematuria with or without pyuria and a positive Bactec test or Versatrek test in urine. The degree of bladder involvement must be assessed by frequency-volume charting, cystography, or even cystoscopy under anesthesia before proceeding with surgical reconstruction. A shrunken bladder may need to be tackled simultaneously by some kind of augmentation.

## TREATMENT

Once the diagnosis of urinary TB is confirmed on investigations, the patients should be put on multidrug antituberculous drugs (ATT). The Centers for Disease Control and Prevention (CDC) and American Thoracic Society recommend that patients with drug-sensitive GUTB should receive multidrug ATT for 6-9 months.[5,6]

Corticosteroids may be given in the initial phase when there is acute tuberculous disease to decrease inflammation/edema and stricture formation.[7] However, its use is not universally advocated.[8] Some cases may have old or quiescent GUTB and have ureteric stricture only and in this setting the benefit of giving ATT for regular duration is questionable particularly if patient is free of general tuberculous toxemia. In most cases, some kind of intervention should be done after the patient has received at least 4-6 weeks of ATT.[3] Every attempt is made to conserve the organs by reconstruction and excise diseased tissue only if mandatory.

## TREATMENT IN EARLY DISEASE

When the investigations show early ureteric disease, every attempt should be made to place a DJ stent. If DJ stent placement is not done, these patients should be followed closely with monthly limited film IVU or renal scan. If there is no improvement or deterioration then early intervention (DJ stenting or Percutaneous nephrostomy (PCN)) has to be done.

## TREATMENT OF ESTABLISHED STRICTURE

For planning of treatment, adequate morphologic delineation by IVU, Retrograde pyelogram (RGP), or nephrostogram and assessment of ipsilateral renal function by Technetium-labeled diethylenetriamine pentaacetate scan (^99m^Tc-DTPA) scan or PCN drainage is mandatory. The management also depends on the overall status of the patient and the degree of involvement of other organs, particularly urinary bladder. The patients of tuberculous ureteric stricture may be grouped into two broad categories:

- *Simple/uncomplicated*: Short segment passable stricture with salvageable renal function and good bladder capacity.
- *Complex/complicated*: Long segment, extensive/bilateral, or impassable stricture with or without salvageable kidney and bladder.

## BILATERAL INVOLVEMENT WITH RENAL FAILURE

In these situations usually one kidney is very poorly or completely nonfunctional. Nephrostomy tubes are placed to decompress the pelvicalyceal system. The individual kidney glomerular filtration rate (GFR) should be calculated and decisions about renal ablation can be taken. Ramanathan *et al*, reported certain preoperative parameters to select patients who will benefit from nephrostomy placement: units with predominantly distal ureteric involvement, good cortical thickness, and a GFR of >15 ml/min.[9] Although PCN is the best available method to assess renal functional recoverability, it is an invasive procedure and sometimes becomes counterproductive, i.e. if the unit cannot be salvaged on account of severe damage, it may require a nephrectomy to avoid a cutaneous fistula.

Once the renal functions stabilize, decision to perform the type of reconstruction will depend on the level of serum creatinine and the degree of reconstruction needed in bladder. These patients are usually young and can tolerate extensive procedures in single stage.

## ENDOUROLOGIC OPTIONS FOR MANAGEMENT OF URETERIC STRICTURE

Many endourologic procedures for management of ureteric stricture have been described in the literature. Although, large series are not present, these procedures may be attempted in select patients. Chantada Abal *et al*, reported good results following endourologic treatment in four cases of ureteric stricture due to TB at follow-up ranging from 2.5 to 6 years.[10] However, it is critical to assess the renal unit for function before starting treatment, as endourologic therapies generally require 25% function of the ipsilateral moiety to have reasonable success rate.[11]

### Double-J stent placement

Double-J stent placement is indicated in two situations: in early disease when there is mild hydronephrosis, DJ stent placement can be done at the time of starting ATT. DJ stent placement also gives the best outcome if the ureteric stricture is short and not dense with a functioning renal unit and with reasonable bladder capacity. Subsequently, this stent can be replaced with a larger size stent. The stent can be kept for a period of 6-12 months and usually by that time the stricture stabilizes. Placement of DJ stent is not always successful. In one series retrograde stent placement was successful in only 41% cases.[9] In a large series of tuberculous ureteric stricture managed endoscopically, Shin *et al*, reported the role of early endourologic management in 77 patients (84 renal units). The renal salvage rate was better in patients who underwent either stent placement or nephrostomy. Spontaneous resolution of stricture was noted in 6 of the 12 renal units that were managed with early ureteral stenting.[12] If the DJ stent placement fails or if the obstruction is getting worse, some type of open/laparoscopic reconstructive procedure is needed.

### Retrograde balloon dilatation

This technique is rarely definitive and usually requires repeated dilatations on a regular basis. The dilatation is repeated every 2 weeks initially, later every 1-2 months, until the upper renal tract has stabilized. Contraindications include active infection and length of stricture more than 2 cm, because dilatation alone will rarely be successful in this setting.[13] This treatment can be done only if it is possible to pass a guide wire across the stricture using transurethral techniques. After dilation a DJ stent is placed. Punekar *et al*, reported the results of retrograde balloon dilation in eight patients of ureteric stricture due to TB.[14] Four failed while four responded favorably. The authors felt that the reason of failure was that the dilation was done after 9 months of receiving ATT. When the authors performed dilatation after 6 weeks of ATT, the response was much better. The authors also felt that tuberculous endarteritis induced ischemia and is the most important adverse factor affecting response to dilatation.[15] Murphy *et al*, used this technique in 80 patients for treating strictures over a period of 25 years and reported 64% success rate.[16] The mean number of dilatations per patient was four. Drawbacks of this technique are high failure rates and repeated procedures requiring anesthesia.

### Antegrade balloon dilation

If retrograde access fails, antegrade approach may be attempted particularly if the patient already has nephrostomy *in situ*. Kim *et al*, attempted this treatment in eight cases and were successful in six.[17] The procedure was aborted in two cases where the guide wire could not be passed across the stricture. In six patients in whom balloon dilation and ureteral stenting were performed, a total of nine stenotic lesions were dilated. Those were four ureteric lesions, two lesions of ureteropelvic junction, two lesions of ureterovesical junctions, and one lesion of calyceal infundibulum. An IVU 9-31 months after the procedure showed improvements both in contrast media excretion and in prestenotic dilatation. Balloon dilation is more successful for patients with relatively short strictures. de la Taille *et al*, reported the use of high-pressure dilatation catheters for the treatment of ureteral stenosis including that due to TB.[18] At a mean follow-up of 8.5 months after stent removal the success rate was 64%. Hoe treated benign ureteric strictures (including those due to TB) by either tapered dilating catheters or angiographic balloon catheters. He demonstrated satisfactory results in strictures especially if treated early.[19] Yip *et al*, reported satisfactory result in a single patient with multiple ureteral and renal infundibular strictures at a short follow-up of 1-year.[20]

### Endoureterotomy

Like balloon dilation, this procedure can be done either through the retrograde or the antegrade approach. The procedure can be performed either under direct vision using ureteroscope or it can be guided fluoroscopically using hot-wire cutting balloon catheter. Richter *et al*, stressed the significance of an intact vascular supply on the success of balloon dilatation.[21] For longer ureteral strictures and those associated with compromised vascular supply, an incisional approach (endoureteromy) was recommended as a more successful endourologic alternative to balloon dilatation.

The position for the endoureterotomy incision is chosen as per the level of the ureter involved. In general, lower ureteral strictures are incised in an anteromedial direction, taking care to stay away from the iliac vessels. In contrast, upper ureteral strictures are incised laterally or posterolaterally, again away from the great vessels.[22] The ureterotomy incision can be made using a cold knife,[23,24] a cutting electrode[25] or laser. The incision should be made from the ureteral lumen out to periureteral fat in its full thickness. The balloon dilation is a useful adjunct either to gain access for endoureterotomy or to wide open the incision.[13] Wolf *et al*, noticed improvement in results by injecting steroids after endoureterotomy.[11]

### Cautery wire balloon incision

This procedure is performed under fluoroscopic control. This procedure is not advocated when the stricture is in close proximity of great vessels, such as at the iliac level of the ureter.[13] As for any form of endourologic management, success utilizing this technique depends on the length and vascularity of the involved segment.[11,26,27] Although results for tuberculous ureteric stricture have not been described, Erdogru *et al*, reported good results in select group of patients using this technique. The favorable prognostic features were: length of stricture <1.5 cm, nonischemic nature of stricture, and adequate renal function.[28]

## SURGICAL OPTION IN MANAGEMENT OF TUBERCULOUS STRICTURE

When endourologic treatment fails in category I strictures or else, in most category II strictures surgical repair of ureteric stricture should be done. In general, procedures described for ureteric stricture due to other benign diseases at the same location can be done. Stricture recurrence in TB is a common complication necessitating close follow-up with regular imaging.[3] Because of fibrosis/loss of elasticity in the dilated segment of the ureter in TB, it may not be possible to mobilize the ureter. As the degree of mobility can be judged only at the time of surgery, the surgeon should keep other surgical options in mind. Today most surgical procedures can also be done laparoscopically,[29,30] though some experience is required to handle the associated fibrosis.

### Ureteroureterostomy

A short defect involving the upper or midureter can be treated by ureteroureterostomy. The anastomosis should be tension-free, hence enough ureteral mobility is essential prerequisite.

### Ureteropyelostomy/ureterocalicostomy

Stricture at pelviureteric junction or upper ureter is uncommon as the renal destruction is so severe in such cases that usually reconstruction is not possible.[4] Pelvic flaps as described for primary pelviureteric junction obstruction are also not feasible due to concurrent pelvic fibrosis. Ureterocalicostomy is preferred as there is often associated caliceal dilatation.

### Psoas hitch

If the bladder capacity is normal, psoas hitch can be performed for small length strictures of the lower ureter. A reflux-preventing technique should be employed whenever possible. When choosing the location for the neocystotomy, one must be careful to avoid any diseased areas of the bladder. Fortunately, the tuberculous infection is mostly localized to the area around the infected ureteral orifice and uninvolved bladder wall can be found.[3]

### Boari flap

If the bladder capacity is normal, Boari flap may be employed for long lower ureteric stricture.

### Intubated ureterotomy

This procedure was popular for long upper and mid-ureteric strictures wherein the incised ureter was left to heal by regeneration over a stent. To support and expedite healing, Naude described the use of buccal mucosal graft in two patients with extensive ureteric tuberculous strictures. They performed simultaneous complex procedures like ureteroneocystostomy, transuretroureterostomy, and Boari flap in the same sitting for reconstruction. In the author's experience, the buccal mucosa takes well and provides excellent results.[31]

### Transureteroureterostomy

If the ureter can be mobilized sufficiently transureter our eterostomy is an option. However, if the other ureter is also diseased this should be attempted with caution.

### Ileal ureteral substitution

For long ureteric strictures involving almost the whole length of ureter or upper ureteric strictures require ileal replacement of ureter. The contraindications include baseline renal sufficiency with serum creatinine value of >2 mg/dl.[13] An isoperistaltic segment of ileum is used and the lumen can reduced by tailoring although Waters and coworkers, in canine study, found no difference in renal perfusion pressure or metabolic derangements in tapered vs. nontapered ileal segments.[32]

### Autotransplantation

For proximal or multiple/pan ureteric strictures when other methods of repair fail or are not feasible, autotransplantation is an option.[33]

## CONCLUSIONS

Accurate delineation of morbid anatomy, ability to salvage renal function by decompression, more effective ATT, greater number of endourologic options, and increasing experience of urologic fraternity have contributed to a favorable outcome in management of ureteric strictures.

## References

[1] (1997). World Health Organization Report on the tuberculosis epidemic.

[2] Qu X, Hou S, Wang X, Huang X, Xu K, Yang C (2000). Middle-aged and elderly patients with hydronephrosis induced by ureteric obstruction: Etiology and diagnosis. Zhonghua Wai Ke Za Zhi.

[3] McAleer SJ, Johnson CW, Johnson WD, Wein AJ, Kavoussi LR, Novick AC, Partin AW, Peters CA (2007). Tuberculosis and parasitic and fungal infections of the genitourinary system. Campbell-Walsh Urology.

[4] Rizvi SA, Naqvi SA, Whitfield HN, Hendry WF, Kirby RS, Duckett JW (1998). Genitourinary tuberculosis. Textbook of genitourinary surgery.

[5] American Thoracic Society, Centers for Disease Control, Infectious Disease Society of America: Treatment of tuberculosis (2003). MMWR Morb Mortal Wkly Rep.

[6] Iseman MD (2000). A clinician's guide to tuberculosis.

[7] Horne NW, Tulloch WS (1975). Conservative management of renal tuberculosis. Br J Urol.

[8] Gow JG (1970). Results of treatment in a large series of cases of genitourinary tuberculosis and the changing pattern of disease. Br J Urol.

[9] Ramanathan R, Kumar A, Kapoor R, Bhandari M (1998). Relief of urinary tract obstruction in tuberculosis to improve renal function: Analysis of predictive factors. Br J Urol.

[10] Chantada Abal V, Gomez Veiga F, Garcia Freire C, Gonzalez Martin M (1993). Tubercular ureteral stenosis: Endourologic treatment of 4 cases. Arch Esp Urol.

[11] Wolf JS, Elashry OM, Clayman RV (1997). Long-term results of endoureterotomy for benign ureteral and ureteroenteric strictures. J Urol.

[12] Shin KY, Park HJ, Lee JJ, Park HY, Woo YN, Tchun YL (2002). Role of early endourologic management of tuberculuos ureteral strictures. J Endourol.

[13] Hsu THS, Streem SB, Nakada SY, Wein AJ, Kavoussi LR, Novick AC, Peters CA (2007). Management of upper urinary tract obstruction. Campbell-Walsh Urology.

[14] Punekar SV, Rao SR, Swami G, Soni AB, Kinne JS, Karhadhar SS (2000). Balloon dilatation of ureteric strictures. J Postgrad Med.

[15] Lang EK, Glorioso LW (1988). Antegrade transluminal dilatation of benign ureteral strictures: Long term results. AJR Am J Roentgenol.

[16] Murphy DM, Fallon B, Lane V, O'Flynn JD (1982). Tuberculous stricture of ureter. Urology.

[17] Kim SH, Yoon HK, Park JH, Han JK, Han MC, Kim SW (1993). Tuberculous stricture of the urinary tract: Antegrade balloon dilation and ureteral stenting. Abdom Imaging.

[18] de la Taille A, Ravery V, Hoffmann P, Hermieu JF, Moulinier F, Delmas V (1997). Treatment of ureteral stenosis using high pressure dilatation catheters. Prog Urol.

[19] Hoe JW (1993). Benign ureteric strictures - Management by percutaneous interventional uro-radiological techniques. Ann Acad Med Singapore.

[20] Yip SK, Peh WC, Li JH, Cheung MC (1999). Case report: Percutaneous balloon dilatation and ureteral stenting for tuberculous renal infundibular and ureteral strictures. Ann Acad Med Singapore.

[21] Richter F, Irwin RJ, Watson RA, Lang EK (2000). Endourologic management of benign ureteral strictures with and without compromised vascular supply. Urology.

[22] Meretyk S, Albala DM, Clayman RV, Denstedt JD, Kavoussi LR (1992). Endoureterotomy for treatment of ureteral strictures. J Urol.

[23] Schneider AW, Conrad S, Busch R, Otto U (1991). The cold-knife technique for endourological management of stenosis in the upper urinary tract. J Urol.

[24] Yamada S, Ono Y, Ohshima A, Miyake K (1995). Transurethral ureteroscopic ureterotomy assisted by a prior balloon dilation for relieving ureteral strictures. J Urol.

[25] Conlin MJ, Gomella LG, Bagley DH (1996). Endoscopic ureteroureterostomy for obliterated ureteral segments. J Urol.

[26] Chandhoke PS, Clayman RV, Stone AM, McDougall EM, Buelna T, Hilal N (1993). Endopyelotomy and endoureterotomy with the Acucise ureteral cutting balloon device: Preliminary experience. J Endourol.

[27] Cohen TD, Gross MB, Preminger GM (1996). Long-term follow-up of Acucise incision of ureteropelvic junction obstruction and ureteral strictures. Urology.

[28] Erdogru T, Kutlu O, Koksal T, Danisman A, Usta MF, Kukul E (2005). Endoscopic treatment of ureteric stricture: Acucise, cold-knife endoureterotomy and wall stents as a salvage approach. Urol Int.

[29] Fugita OE, Dinlenc C, Kavoussi L (2001). The laparoscopic Boari flap. J Urol.

[30] Gill IS, Savage SJ, Senagore AJ, Sung GT (2000). Laparoscopic ileal ureter. J Urol.

[31] Naude JH (1999). Buccal mucosal grafts in the treatment of ureteric lesions. BJU Int.

[32] Waters WB, Whitmore WF, Lage AL, Gittes RF (1981). Segmental replacement of the ureter using tapered and non-tapered ileum. Invest Urol.

[33] Hardy JD (1963). High ureteral injuries: Management by autotransplantation of the kidney. JAMA.

